# What next for local government climate emergency declarations? The gap between rhetoric and action

**DOI:** 10.1007/s10584-021-03147-4

**Published:** 2021-08-01

**Authors:** Candice Howarth, Matthew Lane, Sam Fankhauser

**Affiliations:** 1 Grantham Research Institute on Climate Change and the Environment, London School of Economics and Political Sciences, London, United Kingdom; 2 School of Geosciences, University of Edinburgh, Edinburgh, United Kingdom; 3 Smith School of Enterprise and the Environment, University of Oxford, Oxford, United Kingdom

**Keywords:** Climate emergency, Local scale, Urban governance, Climate change, London, United Kingdom

## Abstract

The UK, like other countries, has seen a proliferation of declarations of local climate emergencies. While these declarations have been interpreted as a demonstration of ambition, little is known about how and why they actually came about when they did and the implications this will have for what happens next. Focusing on London, UK, we present evidence collected via semi-structured interviews with experts and practitioners involved in the propagation of climate emergency declarations to critically explore how and why these declarations emerged, and the various different roles they are perceived to play for different local actors. Our findings reveal four journeys to local government declaration of a climate emergency (made actively from above, passively from above, actively from below, and passively from across) and three interwoven purposes (statements of intent, acting as a political gesture, and stimulating local action). We argue that these three purposes combine and coalesce to correlate the declaration of climate emergency with a local responsibility for emissions reduction, leaving little analytical space to question the scalar disconnect between the immediacy of the narrative at local scales and the slow-burning (and) global nature of the threat in question. If these emergency declarations are to be an opportunity for change in the governance of climate change, then the question of ‘what next?’ requires more in-depth, thorough and constructive engagement with the type of climate action the declarations are expected to induce while considering how this aligns with existing responsibilities and resource bases of local government.

## Introduction

In this paper, we engage with the phenomena of climate emergency declarations, which have been enacted by local actors across the world since 2016. Both research and practice on climate change governance at the local scale have been energized by the widespread declaration of an emergency state at multiple levels (e.g. states, companies, museums, universities). Despite the time that has passed since the first climate emergency declaration (in Darebin, Australia in December 2016), these declarations are still predominantly framed as the starting gun for local action on climate change, through an emergency form of climate governance, intended to move us away from ‘business as usual’ approaches (Davidson et al. 2020; Sutton 2017). Indeed, off the back of local declarations, a number of ‘place-based’ climate and sustainability strategies (or ‘climate action plans’) and stakeholder networks are beginning to emerge as a way of expanding the capacity of local government while simultaneously holding them to account with regard to their own climate action and emission reduction plans (see Creasy et al. 2021 for a UK account). However, a closer look at the political context of climate emergency declarations at the local scale suggests that they are themselves part of a wider process (rather than merely a starting point for place-based action), and here we examine the political, cultural and institutional landscapes from which local emergency declarations have been spawned.

The attributes of local climate emergency declarations stand in stark contrast to other invocations of emergency governance (such as the response to the COVID-19 pandemic), wherein powerful national governments move to suspend and curtail citizen rights and freedoms. In many respects, climate emergency declarations are themselves a manifestation of those freedoms and rights. We therefore frame anthropogenic climate change as a ‘slow emergency’, and we explore how the nature of this particular ‘emergency’ is being understood at the confluence of both ‘slowness’ and ‘localness’. Our focus is on London (UK) and its 33 municipalities (32 boroughs plus the City of London). The climate debates in London’s boroughs are in many ways typical of the local governance dynamics in other UK cities and around the world, for example, in terms of party politics, local delivery capacity and multilevel governance. In London, as elsewhere, the role of ‘local councils’ is very much nested within a hierarchical set of government ‘levels’ across which ambitions for action on climate change may or may not be aligned. We explore how the proliferation of declarations at the local scale is to be understood and the implications this might have for what comes next. For example, we consider if these are predominantly a cry for help by local authorities or a commitment to ‘do their bit’ in the fight against climate change.

In the next section, we unpack the language used with regard to the climate emergency in order to problematise the seeming disconnect between the scale of the emergency (global) and who is declaring a commitment to address this (local governments). The subsequent methodology section (Section 3) explains how this framework informed the collection of data through stakeholder interviews in London. Section 4 presents the first topic to which we make a contribution: an analysis of the ways in which these declarations emerged from their particular contexts. Section 5 then presents findings regarding the perceived purpose of these declarations, derived from the expert opinions solicited. Our concluding section (Section 6) then reflects on how the paper’s findings add significant depth to our understanding of what might come ‘next’ following a declaration, before pointing to avenues for future research.

## Climate change and emergency governance regimes

### The global landscape of local climate emergency declarations

The first climate emergency declaration in Darebin in 2016 was followed by declarations in the US cities of Hoboken, New Jersey (April 2018), and Berkeley, California (June 2018), which in turn set the precedent for a wave of worldwide declarations in 2019. While there is limited literature that discusses the effectiveness of these early emergency declarations as tools for local climate action, Darebin council’s invocation (in their declaration text) of the fact that ‘we’ are in a state of climate emergency offers an important conceptual starting point. Who is the ‘we’ in question here? And what role is being played (intentionally and unintentionally) by such modest, local actors provocatively sounding the alarm on global climate change?

The language used to address climate change has been constantly changing over the past two decades, reflecting both an evolution in the perceived urgency of the challenge and a grappling for the right way to represent it as an urgent problem needing to be solved. The emergence of ‘climate crisis’ and the subsequent prevalence of ‘climate emergency’ is an example of this. Notably, there was a surge in the use of the term ‘climate crisis’ between 2006 and mid-2008 worldwide (much less pronounced in the UK), and the use of ‘climate emergency’ rose significantly from mid-2019 onwards, particularly in the UK. Illustrating its rise to prominence, ‘climate emergency’ was named the Oxford English Dictionary’s word of the year in 2019 when its use rose by 10796% (Zhou 2019). Their definition of a climate emergency is:

a situation in which immediate action is needed to reduce or stop climate change and prevent serious and permanent damage to the environment. (Oxford English Dictionary 2020)

According to Spratt and Sutton (2008), this movement to ‘emergency’ language is likely due to the fact that emergencies are a form of crisis requiring action well beyond business as usual. Despite over 40 years of warnings from scientists, there has been widespread failure to address climate change (Ripple et al. 2020). Spratt and Sutton (2008) thus highlight that the use of emergency language is the start of a movement towards more drastic action, accurately predicting that this would climax in the formal climate emergency declarations of governments. Similarly, in considering the legal implications of a ‘climate emergency’, Lindsay (2010) argues that emergency language implies the formal use of emergency government or ‘state of emergency’ to tackle the issue. Hence, an emergency is characterised by recognition that a threat is large enough to demand a mobilisation of resources and requires the engagement of governments to coordinate and administer a rapid response. Declaring a sustainability or climate emergency signals that governments and communities need to cooperate and act with speed, on a large scale (Spratt and Sutton 2008). ‘Emergency’ discourse therefore enables the communication of the sense of urgency with which we must act in response to the inaction we have seen preceding this language shift.

At the international scale, the IPCC’s, *Global Warming of 1.5°C* ( 2019a) report is often considered to be the catalyst for the explosion of emergency declarations in 2019 although it makes no explicit reference to ‘climate emergency’ or ‘crisis’. However, this report was widely used as a platform for catalysing an emergency response to the threat of climate change. For example, Extinction Rebellion frequently cites the report in their factsheets for supporters and media campaigns (Extinction Rebellion 2018a, 2018b). The IPCC meanwhile avoid using emergency rhetoric in order to align with their aim to refrain from being politically prescriptive, instead of describing climate change in terms of risk. The 2018 UNEP Emissions Gap Report played a similar role (Allen 2019; Asayama et al. 2019; Cretney and Nissen 2019; Hulme 2019; Rode 2019); however, while unofficial publications have consistently adopted an ‘emergency’ rhetoric (UNEP 2019; 2020a, 2020b, 2020c), official publications made by UNEP avoid the term. This could reflect the different audiences that UNEP’s official and unofficial publications are aimed at, for example, policymakers in the former and concerned citizens in the latter. Unlike UNEP, the IPCC has also avoided using ‘emergency’ rhetoric in unofficial publications (e.g. blogs, news articles), with the most demanding call for action being calls to ‘urgently’ reduce greenhouse gases (IPCC 2019b).

The relationship between scales of authority and the commitment to (a) political interpretations of climate change and the threat posed has important implications for how we understand proposed ‘solutions’ subsequently put forward. The question we must ask is where does the lack of a requisite ‘global’ authority (able to be mapped onto the scale of the challenge posed by climate change) leave local authorities?

### Framing local declarations of a global emergency

In the past two decades, global governance of climate change has become increasingly fragmented and decentralised, taking the form of a ‘regime complex’ made up of numerous loosely coupled regimes (Keohane and Victor 2011). Consequently, action taken against climate change must be examined with respect to the various different contexts that they operate in, namely: international organisations, national-level governance and local-level governance. Differentiating between parts of the climate regime along these lines is by no means final or clear cut. Furthermore, the multilevel governance literature shows that regimes are often overlapping and actions in one regime do not occur in isolation of the others (Di Gregorio et al. 2019). With the 2015 Paris Agreement setting a precedent for this increasingly polycentric approach (Jordan et al. 2018), actors at the subnational level (with an emphasis on cities and their governments in particular) are expected to play a greater role in climate governance (Van der Heijden 2018).

Despite this increasing emphasis on sub-national engagement with climate change (Schlosberg et al. 2017; Sotto et al. 2019), the invocation of an emergency discourse at this scale raises a number of questions. Firstly, a great deal of theoretical work has been done to unpack the way in which a discourse of ‘emergency’ is drawn upon by governments to justify interventionist decisions which circumvent normal democratic checks and balances during times of ‘exception’ (Agamben 2008). To date, however, this has principally focused on national-scale governments with the ability to wield substantial resource bases in the face of external ‘threats’ (Bandt 2009; Calhoun 2010). Secondly, these resources are generally wielded in order to mitigate the risk posed by perceived threats to the sovereignty of the government in question (Humphreys 2006). When it comes to anthropogenic climate change, then, and its global nature and seemingly existential threat to life on Earth, it is difficult to create the requisite separation between the threat and the threatened. Rather than having spatial characteristics with the potential to undermine sovereign states, climate change has temporal ones, threatening populations of the future. As a result, local emergency declarations must be situated in the broader landscape of the shifts in governance required to deal with the unique nature of the threat. However, while the requirement for action has become normalised, there has been little progress in articulating what exactly those actions will look like and who will be responsible for driving them.

The traditional notion of what counts as an emergency has recently been broadened with the concept of the ‘slow emergency’ (Anderson et al. 2019). This concept shares similarities with the political economy concept of ‘slow violence’, whereby environmental damage causes insidious, slow-burning social harms, often against the poorest and most marginalised communities (Nixon 2011). Appreciating the ‘slow’ nature of emergencies aims to shift academic study of emergency from how life is governed towards the emergency claims and acts made by marginalized people from within situations of ‘attritional lethality’ (Anderson et al. 2019, p627). Anderson et al. argue that emergencies are a way that communities, including environmentalists in the 2019 climate protests, render the impacts and effects of slow emergencies as sensible and accountable (p634). While the timescales in question help us appreciate the value of framing anthropogenic climate change as a ‘slow emergency’, the effects of which are both nascent and distributed in complex ways, this raises a number of issues regarding the *governance* responsibilities associated with such emergencies.

The local nature of declarations of a planetary-scale emergency, as well as the way in which such declarations have proliferated across the world via local governments before being embraced at the national and international levels serves to demonstrate what Castan Broto (2020) describes as the ‘messiness’ of climate governance. With both an appreciation for the severity of the climate challenge and the will to do something about it so politically fragmented, we might see it as inevitable that such an ostensibly grand challenge was able to trickle down to the smallest scales of government before finding a voice. Much of the existing literature on local and urban climate governance, however, remains cautious to narratives of local climate saviours stepping into a national and global action vacuum, questioning the validity of such claims against the backdrop of dominant political-economic regimes (Angelo and Wachsmuth 2020; Van der Heijden 2019).

In the context of the UK, such reservations seem well founded given recent commentary on the failure of the national government to adequately devolve the required resources in the post-declaration era (Green World 2020). Furthermore, as argued by Castan Broto (2020), narratives of action against climate change rooted in particular places sit uneasily with the seemingly ‘mobile’, place-agnostic nature of existing climate change policy mechanisms. Perhaps, such as the recently proliferated emergency declarations, which, as we have discussed here, have travelled between and through local jurisdictions around the world and which—through the case of London— will now form the focus of the paper’s empirical contribution.

## Methodology

Two studies have sought to further our understanding of local government climate declarations. The first, by Harvey-Scholes (2019), examined local governments in the UK that had declared climate emergencies and their plans or commitments to mitigate against and adapt to climate change. A correlation was found between the councils that had declared climate emergencies and how ambitious or detailed their climate change mitigation plans were. The study demonstrated varying levels of integration, ambition and detail between different council’s climate emergency declarations. The second, from Davidson et al. (2020) examined two case studies (including Darebin) in order to better understand how declarations were being operationalised through emergency plans. The authors develop a ‘climate emergency response attributes framework’ in order to interpret the plans. We seek to contribute to this early body of knowledge, by drawing on semi-structured interviews with those involved with local emergency declarations in order to offer further analytical depth to understanding how these emergency declarations have come about and, most importantly, how this might affect the question of ‘what happens next’ at the local level.

**Figure 1.**
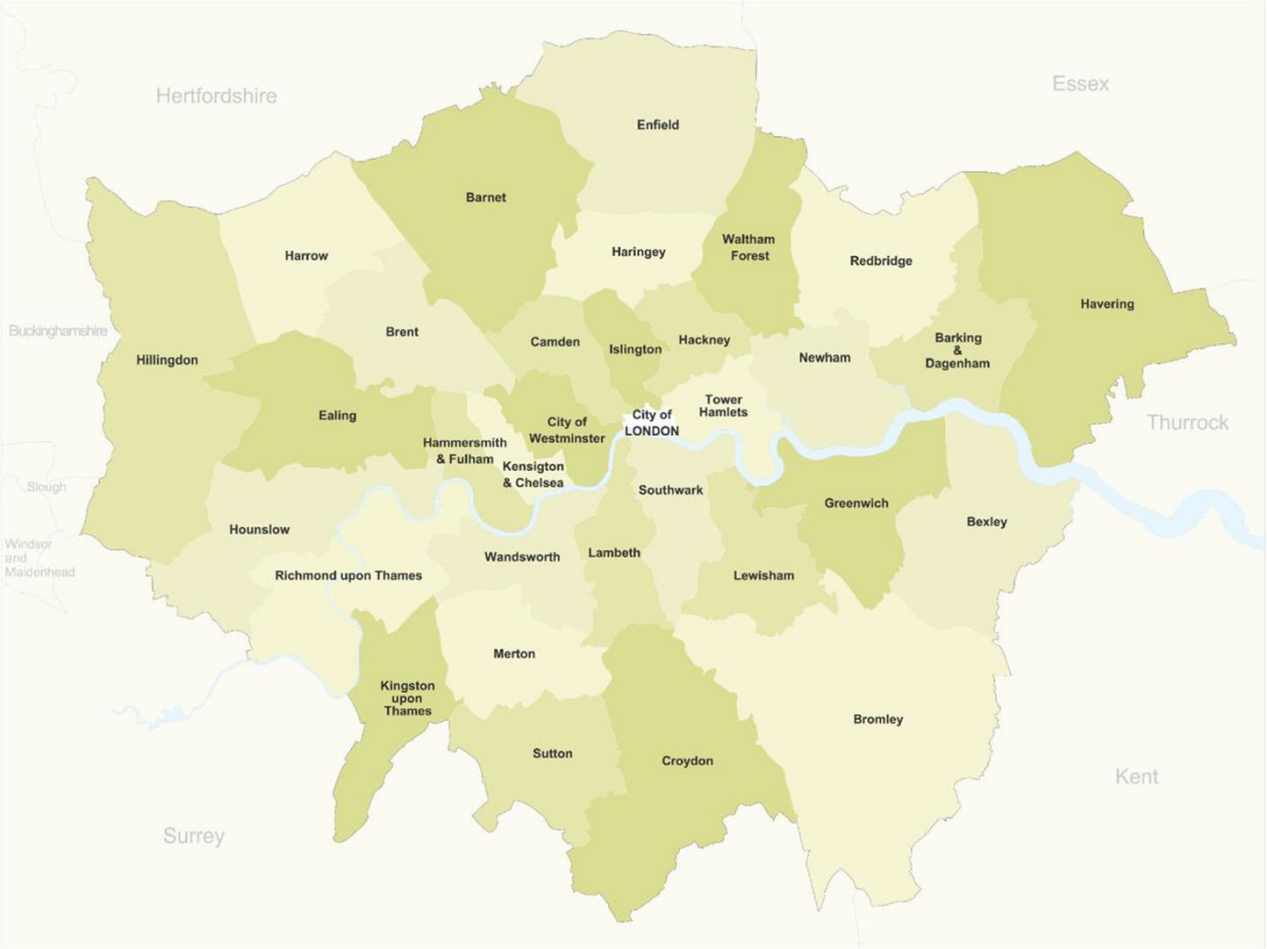
Map of London Boroughs, from https://map.comersis.com/

Our empirical focus in this regard is drawn from a case study of London, UK (Figure 1). The Greater London Authority (GLA), the devolved regional governance body of London, declared a climate emergency in December 2018 with a target to make London a carbon-neutral city by 2030, and London borough councils have (mostly) followed suit with their own individual net zero targets. In addition to providing a location where a number of local authorities have declared climate emergencies, this focus on London also allowed us to incorporate some comparative reflections on those local boroughs who have decided *not* to declare a climate emergency and what this tells us about the use of emergency declarations as a political tool vis-à-vis both horizontal (inter-local government) and vertical (with national government) relations. The research aimed to better understand what local climate emergency declarations (CED) actually *are*, by examining (i) how they have emerged (and the contexts from which they have emerged) and (ii) what purpose they serve for the declaring authorities. Having framed climate change as a ‘slow emergency’ in the previous section of the paper, our focus on these two topics is premised on first investigating how the ‘slowness’ of the emergency in question maps onto the speed in which these declarations were conceived of and subsequently enacted. Our interest in the second topic of ‘purpose’ is subsequently about analysing the extent to which these declarations ‘fit’ with the traditional roles and responsibilities of local government and, in doing so, diverge from a more ‘traditional’ understanding of emergency governance premised on the curtailment of freedoms and democracy in order to directly address the impending threat. By building up a picture of how these declarations emerged and how their role in the world is being understood, our ultimate aim is to inform how the question being asked by many should be framed: what next?

To identify experts to interview, an initial stakeholder mapping exercise was conducted of experts from academia, policy and civil society in the UK, with knowledge and experience of climate emergency declarations in the UK with a local focus on London. Through this process of expert elicitation, sixteen semi-structured interviews were carried out, using purposive sampling, with 5 UK academics (labelled as ‘Aca’), 6 policymakers or representatives of policymakers/policy organisations (‘Pol’) and 5 civil society organisations (‘Civ’). Interviewees covered a range of backgrounds and expertise with specialisms including climate governance, climate democratic deliberation, city governance, climate activism, climate policy and decision-making, with varying levels of seniority to ensure a representative sample. Organisations represented by interviewees included universities, UK local and national government departments and networks, city-based climate initiatives and civil society organisations.

Interviewees were asked a series of questions on UK climate emergency declarations (see Table 1) based on the two focus themes of (i) emergence (how did the declarations arise, and the balance of relationships between other local authorities and with national/regional governments) and (ii) purpose (what are the declarations for and how are they perceived). Interviews took place in the UK in person or over the phone, with questions piloted beforehand. Each interview lasted 30–60 minutes and was audio-recorded, and data was transcribed and analysed via the software NVivo using a combination of deductive and inductive thematic analysis. In the case of our interest in CED ‘emergence’, we analysed the data deductively in order to identify the most dominant backstories helping to explain the emergence of the declarations in their particular contexts. For the question of purpose, we took a more inductive approach to identifying the multitude of purposes covered by the interviewees. Where possible, we have quantified these themes by specifying which/all the interviewees that made reference to them; however, where we have specified a single interviewee, these represent salient examples of a point of view, and do not mean only one interviewee referred to it. The sample size (N=16) and UK representation of the participants, while suggesting a need for further research with larger sample sizes from different geographical and sectoral populations, is deemed sufficient to capture a diversity of views, given the context, scope and research question. In what follows, we first present four identified back-stories or ‘journeys’, which answer the question of how local CEDs in the context of London emerged (Section 4.1). In order to draw out these backstories, we analysed the data with the ‘slow emergency’ perspective in mind: (i) what agency did the local authorities have in making the declaration at this time; and (ii) what was the relationship to other scales of governance. We subsequently present the findings of the more inductive, thematic, analysis of the data on CED *purpose.* This is delivered in a more speculative manner, capturing the wide range of interviewee responses to each question. As mentioned above, the four questions asked here tie directly to the notion of ‘slow emergency’ and the agency that local government has (or does not have) in responding accordingly to the unique nature of this particular challenge.

**Table 1.** Interview themes and questions

Theme	Interview questions
Emergence of the CEDs	What is your take on the CEDs? What was their story in London in 2019? Why do you think CEDs have come about? How were the declarations perceived?
Purpose of the CEDs	Are they effective ways of enhancing climate action? Why do you think some boroughs have chosen not to declare a climate emergency? What is missing from the CEDs? What needs ‘watching’ now that the declarations have been made?

## Emergence of the climate emergency declarations in London

**Figure 2.**
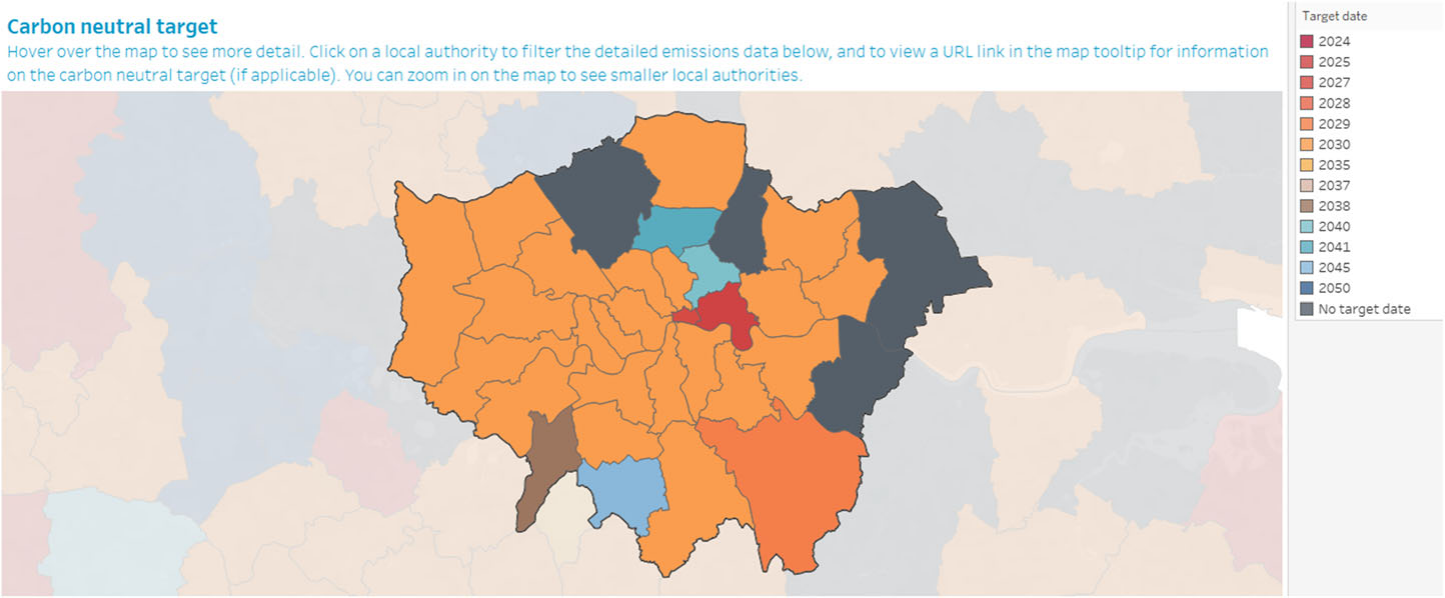
Map of London boroughs with their climate emergency declaration-related net zero targets. Used, with permission from Aether-UK

Between January 2019 and January 2020, 28 of the 32 London councils (excluding the Greater London Authority) declared a climate emergency. Most declarations are associated with emission reduction targets, ranging from 2025 (e.g. Tower Hamlets) to 2040 (e.g. Hackney), and the majority of councils setting a net-zero target of 2030. Just five councils (e.g. Camden and Waltham Forest) have failed to specify a net-zero date (see Table 2 and Figure 2). The council motions appear homogeneous, suggesting a template (Climate Alliance 2019) may have been followed: noting the issue of climate change, highlighting the IPCC target of 1.5°C, sometimes considering the costs inflicted by climate change to the borough, a list of actions already taken by the borough, acknowledgements of the role of national government and cities in addressing climate change, followed by a resolution to declare a climate emergency and their additional commitments including calling on the government to provide resources. The similarity in the declarations may also result from councils copying each other or basing their declarations of the first declaration in Lambeth in January 2019.

**Table 2.** Climate emergency declarations in London councils (at end of 2020)

London council	Party	Date declared	Net zero target	Who declared?	Adaptation mentioned?	Citizen engagement?
Barking and Dagenham	Labour	Jan 20	2030	Council motion	No	n/a
Barnet	Conservative	Undeclared	None	n/a	Undeclared	n/a
Bexley	Conservative	Undeclared	None	n/a	Undeclared	n/a
Brent	Labour	Jul 19	2030	Council motion	No	Yes
Bromley	Conservative	Undeclared	2029	n/a	Undeclared	n/a
Camden	Labour	Apr 19	None	Cabinet member	No	Yes
Croydon	Labour	Jul 19	2030	Council motion	No	Yes
Ealing	Labour	Apr 19	2030	Council motion	No	No
Enfield	Labour	Jul 19	2030	Council motion	No	No
Greater London Authority		Dec 18	2030	Mayor	No	No
Greenwich	Labour	Jun 19	2030	Council motion	Yes	No
Hackney	Labour	Jun 19	2040	Council motion	No	Yes
Hammersmith and Fulham	Labour	Jul 19	2030	Council motion	No	Yes
Haringey	Labour	Mar 19	2030	Council motion	No	No
Harrow	Labour	Jul 19	2030	Council motion	No	No
Havering	Conservative	Undeclared	None	n/a	Undeclared	n/a
Hillingdon	Conservative	Jan 20	2030	Council motion	No	No
Hounslow	Labour	Jun 19	2030	Council motion	No	Yes
Islington	Labour	Jun 19	2030	Council motion	No	Yes
Kensington and Chelsea	Conservative	Oct 19	2030	Council motion	No	No
Kingston-upon-Thames	Liberal Democrat	Jun 19	2038	Council motion	No	Yes
Lambeth	Labour	Jan 19	2030	Council motion	No	Yes
Lewisham	Labour	Feb 19	2030	Council motion	No	No
Merton	Labour	Jul 19	2030	Council motion	No	Yes
Newham	Labour	Apr 19	2030	Council motion	No	Yes
Redbridge	Labour	Jun 19	2030	Council motion	No	No
Richmond-upon-Thames	Liberal Democrat	Jul 19	2030	Council motion	Yes	Yes
Southwark	Labour	Mar 19	2030	Council motion	No	Yes
Sutton	Liberal Democrat	Jul 19	2030	Council motion	Yes	No
Tower Hamlets	Labour	Mar 19	2025	Council motion	No	No
Waltham Forest	Labour	Apr 19	None	Council motion	No	No
Wandsworth	Conservative	Jul 19	2030	Council motion	No	Yes
Westminster	Conservative	Sep 19	2030/40	Council motion	No	No

Through analysis of the interview data, we identified four important drivers for the declarations made by the different London borough council, steered by forces operating: (i) actively from above (devolution); (ii) passively from above (opposition); (iii) actively from below (community activism); and (iv) passively from across (inter-local competition and diffusion).

### Actively from above

While there is variation in targets across the Boroughs, there is recognition from the GLA of public and business support for more ambitious targets, seen as further validation for the London Mayor’s target for London to be a net zero city by 2030. There are two important ways in which the decision to declare a climate emergency by London Boroughs was influenced by the active involvement of the higher ranks of power. The first is through devolution. While devolved responsibilities to local government are particularly weak in the UK when contrasted with other parts of the world, in the case of London, a greater sense of autonomy has trickled down to boroughs.

if [the Prime Minister] declared [an] emergency it’s through the councils, there’s a vote on this, so it’s democratically endorsed. It’s not just the Dean of the Borough, or the Mayor, and you are in some ways collectively giving recognition to the problem. And that’s the first step*.* (Aca1)

While beginning to take root in other parts of the UK, London’s mayoral status in particular was pointed to as an important factor in setting the agenda (Aca1; Aca2).

the declaration is important, and it is probably easier than doing anything else, but it isn’t that easy to get it unanimous. So causing ructions and splitting councils can actually be counterproductive, so it has to be done in a diplomatic listening way. (Civ5)

I think in some cases there will be like a political policy entrepreneur, or leadership or leaders, that will be able to go beyond what is their legal mandate for them. (…) It will be interesting to know what is the specific plan they have, they’re talking about, and I can see how that relates to the 1.5 at least of mitigation. (Aca3)

The second important way in which a motivation to make a climate emerge declaration is manifested from above is through party political allegiances. While a large number of conservative councils *have* declared an emergency and have subsequently aligned their targets with the national government’s net zero 2050 target (Pol5), it is ‘mostly Tory councils that haven’t declared’ (Civ4). Out of the 32 London boroughs, four have not declared a climate emergency and are conservative-led: Barnet, Bexley, Bromley and Havering (see Table 2). For Barnet, Bexley and Havering, council motions to declare were proposed but were dismissed or voted against in July 2019, with a Barnet councillor noting that Parliament’s national declaration was sufficient and that Barnet faces the same challenges as the wider UK (Allin 2019; Roach 2019). Bromley has taken a different stance, stating that there is a climate emergency regardless of their declaration and that they are taking action through making a net zero target of 2029, rather than declaring mere words (Bull 2019).

Probably political make-up will influence that, I think I mean we will probably find that those are more likely to be Conservative councils, but they’re not necessarily just that, I think there will be some concern around just transition, and the fact that action on climate emergency could mean, or could be perceived to mean slowing down of industries of employed people. (Civ3)

### Passively from above

For those boroughs that do not have conservative majorities, local scale declarations offered an opportunity to enact an opposition movement and draw attention to the inadequacy of leadership on climate change at the national level (Aca2).

So there was that confluence of events, and then the Green Party motioned and some Labour motions as well, that just set the ball rolling really. The biggest surprise to me, I suppose, was winning this at so many local authorities pass motions, not declaring a climate an emergency because the evidence debate for that is, has been strong for the last 30 years. (Civ2)

All of these declarations happened, I think very much driven, I think Labour, Green, Lib Dem councillors very much pushing it, but some Conservatives as well were. (Civ3)

While in part tethered to an opposition to national government however, local declarations also offer an opportunity for local authorities to more firmly situate themselves as important players at the place-based scale, transition from simply being service providers to becoming more pro-active, proprietary agents:

I think we’ve come back to a place where some real hard-hitting facts have been given to us. And councils, as leaders of their local place, have, are building on their role that they’ve been playing to sort of put this more at the forefront of a local place’s agenda. (Pol4)

### Actively from below

The most commonly cited motivator for the declaration of climate emergencies by London borough councils identified by interviewees related to the role of civil society and particularly to activism within local communities (Aca2-3; Aca5; Civ1; Civ3-4; Pol1-4; Pol6).

there have been councillors in most of the councils who have picked this up and run with it. But they have been able to get the declarations through because there’s clear public support, you know, that most of the council meetings where these have been successful, the public galleries have been packed. (Pol2)

Over half of the declaring boroughs appear to have continued to engage citizens in the process, for example, through an assembly or other means, since declaring an emergency (e.g. Brent, Croydon and Newham). Again, this activism becomes intertwined with the political affinities of both local authorities and their communities:

…a lot of the pressure will not come from officers but it will come from political activists (...). Many of them will be interested in the environment and they will have close links to the activists who make up the basis of say the Labour party in the London borough concerned, particularly the Labour party. (Aca2)

A number of interviewees emphasised the close relations between activist movements and local political groups, from which the process snowballed (Civ2; Aca3).

I think that the way that the emergency declarations were made was very strongly influenced by the external pressures of the social groups. And I think that’s in the moment in that context, the councils are quite receptive to these suggestions and probably one political party or one member of the council took the proposal and put it forward. (Aca3)

Similarly, the importance of youth voices in building momentum to declare climate emergencies was highlighted by interviewees (Civ2-4; Pol 2):

... climate change was much higher up the political agenda in young people’s viewpoints than perhaps the older generation. So there’s kind of a confluence of different events that were shoving climate change up the agenda, which meant, (...) more a spark to a kind of dry kindling. (Civ2)

The role played by the interwoven nature of scales is key; while there may be questions over the power local government holds to enact policy change, it provides a powerful interventionist voice behind which weight can be thrown, particularly when the voice is amplified through sheer numbers:

by Spring 2019, (...) everybody was talking about 1.5 degrees. And then that came together with Extinction Rebellion, who were really live and pushing debate on the street. And then the youth strikes, (...) the March and April demonstrations were massive, that’s when they broke into the public consciousness. And at that point, that’s when the government started to declare, and then there was a critical mass, (...) they started creating a website and branding around declaring the climate emergency, and there was a big network emerged in the UK and beyond. (Aca5)

### Passively from across

The final point in the previous section makes reference to the institutional tissue of emergency declarations which emerged within and between the scale of local government. Our final form of motivator towards declarations builds on this, drawing attention to the intergovernmental nature of local action as steer diffuses out from the actions of other local boroughs.

Camden declares a climate emergency and starts holding citizen juries or focus groups, you can be sure that other Labour boroughs and then some progressive Conservative boroughs will follow suit because they want to be seen to be in the pack and not behaving - there will be some on the other side who think this is not for us. (Aca2)

they happened very quickly and there was a bit of a copycat thing, a feeling that well, my neighbouring borough has done it, we have to do it. I don’t think that a lot of thought went into coming up with the timelines. (Civ3)

The ‘domino effect’ (Pol5) of emergency declarations served to usher in a certain template for declarations:

I don’t think a declaration is a tool for action. The plan should be their tool for action if it’s done properly. So we know, and I know from having come out of the consultancy world, this is a really good earning opportunity for them [consultants]. And some of them are earning huge amounts of money by just churning out the same plan over and over again. (Civ5)

The language used in some of the declarations suggests an element of competitiveness between boroughs, with many expressing their desire to be the greenest borough, such as Barking and Dagenham aiming to be the ‘green capital of the capital’ (Burford and King 2020). Many boroughs, including Croydon, Richmond-Upon-Thames and Hackney, expressed similar ambitions, with Hackney stating their declaration is ‘most robust’ (Hackney Council 2019). This appears to highlight pressure amongst councils to keep up with each other and declare an emergency, although information on this is highly dependent on availability and accessibility of evidence from each Borough council (e.g. suitable website although absence of a website does not mean absence of action).

## Purpose

### Effectiveness in encouraging action

The interview data highlighted a variety of themes which shed light on the extent to which these CEDs are effective in encouraging action. The declarations are driven by the question of what their backstories suggest about what may come next and the CEDs are seen as (i) a statement of intent, (ii) acting as a political gesture and (iii) stimulating local action. Returning to the very start of the paper, in order to shed light on what might come next after the declaration of an emergency, the question remains: who are these declarations for? Having used the local scale to raise the alarm, who is it that is supposed to respond and what tools are required for the job?

The CEDs are seen as a statement of intent by many of the interviewees (Civ4; Aca2-3; Aca5; Civ1; Pol6) without necessarily providing a strategy for taking action with appropriate tools highlighted for implementing them.

At the end of the day, these climate declarations are statements of intent but they’re not a framework for taking action. I think they should, of course, kick off a local framework but I think, in most cases, and probably the exceptions to a certain extent may be some of the big cities, but even then there are limits. But unless there is a national central government framework that gives more power to local authorities, gives more direction to local authorities, gives them more resource, I think the statement of intent just simply can’t be delivered. (Pol6)

it’s a statement of intent and you need legislation, policy and investment to flow from that. And unless those three things are put in place, then again, nationally or locally, they’re just statements of intent. But (...) at the very most basic levels of that, one of the components of that kind of framework, the policy framework, is there’s no precise understanding about what emissions local authorities produce, what exactly they’re able to deliver. There are a number of different ways that authorities across the country are measuring things. (Pol6)

Similarly, Aca1, Civ1, Civ3, Pol3 and Pol5 expressed a concern that the declarations are merely a political gesture reinforcing the previous theme of CEDs being a statement of intent resulting from scepticism as to what can be delivered in practice.

I think in our case it’s because we think that is both a political gesture and our job is substance more than gesture, is probably the summary of it. We talk about the climate emergency a lot, including the language that we now systematically use and is used by our chief executive and chair, and we think that is appropriate, but declaring a climate emergency is from our perspective a political gesture. (Pol3)

I think many of those levers reside with central rather than local government, although I think local government probably has more levers in relation to adapting to climate change as often as it does. I think when people declare that they’re going to achieve net zero in the city, I am slightly sitting there scratching my head because the city authority just doesn’t have the levers to do that. (Pol3)

Nevertheless, they are seen as effective ways of stimulating and bringing together local action (Aca3; Civ3; Aca5; Pol3), many of them having resulted from a surge in local grassroots and civil society pressure to make the declaration.

So at the end it’s also really important that these grassroots organisations and other organisations are a success. (...) so they should be able to follow up on that guarantee or promote or supervise, the [policy changes]. (Aca3)

And it is essentially then the argument that (...) was brought to the street and where then the question is, alright, how do you respond to at least a perceived need of far more dramatically reduced carbon emissions than before, and also a clear perception that the tools and all the efforts which are underway are unlikely to deliver. And as a policymaker at the urban level, you don’t have a lot of power, but you have one amazing tool, which we now are seeing rolled out in front of our eyes, which is around emergencies. (Aca1)

some councils are really good at joined-up working, at collaborating across the council and then beyond the council (...). Other councils are so locked in their traditional silo working; that is a massive barrier [of] implementing, well, creating the Climate Action Plan in the first place and then implementing it. (Civ5)

### The decision not to declare

Rather than succumbing to emergency rhetoric, some local authorities are taking a different approach to the challenge without declaring a climate emergency but still thinking and delivering climate action in their own way (Aca5; Civ1):

in some cases, they’ll probably end up setting new targets or publishing new strategies but are just not going to use the emergency language. I think I would say pretty much every single borough is doing more than it was. Some are doing vastly more. Some from a place where they were doing nothing are doing a bit, which is proportionately a much bigger increase. (Pol5)

The word emergency certainly didn’t [help], for some conservative councils, I think they saw it as a bit hyperbolic, they could see they needed to do stuff around climate change, they saw this as a bit of a bandwagon, they were reluctant to jump on it and shout emergency. (Civ2)

Nevertheless, there is recognition of national government leadership on the climate emergency and the national net zero target by 2050 implying there is less of a need to go any further with the alarmism:

They probably have senior leaders in cabinet who are insisting on having a very strong evidence basis before they make any declaration of sorts and I think that’s really sensible. (…) Regardless of whether you declare a climate emergency or not, that is the legal default already, so you declaring it or not is irrelevant in some sense, because you have to get there, legally speaking. Perhaps that’s what the local authorities who haven’t declared an emergency are thinking about as well, like net zero 2050 is ambitious enough and we are legally bound to meet that, (...) as a nation we have that target and local authorities will have a responsibility there. (Civ1)

I wonder if others haven’t done it because then it could hold them to doing something about it. Because if these boroughs actually decarbonised by 2030 that’s like a super massive ask and none of them are actually doing anything like what’s commensurate with it really. (Civ4)

### Gap: adaptation versus mitigation focus

One of the main gaps in the climate emergency declarations that emerged from the interview discussions was a lack of focus on adaptation in comparison to mitigation. The notion of a climate emergency is primarily associated with the urgent need to reduce greenhouse gas emissions by a certain target date (often, 2050) in order to stay within 1.5 or 2°C warming, mirroring national action on the climate emergency. As a result, climate action has often focused on mitigation. However, the focus through which the climate challenge reveals itself to local governments as something tangibly ‘new’, which requires an overhaul of how it carries out its existing operations, is through *both* adaptation and mitigation. The majority of declarations focus predominantly on mitigation and fail to mention adaptation, while a small number have considered it (e.g. Sutton) in subsequent action plans, notably regarding flooding (e.g. Greenwich, Richmond-upon-Thames and Sutton).

All of [the declarations] have this kind of plea to government for more powers and funding at the end of them and that seems to be the kind of crux of what holds most councils back from actually implementing their big ambitions which are usually to like decarbonise by 2030. (Civ4)

So it’s very much seen as a carbon emission reduction strategy. (Pol5)

Adaptation is folded into a longer-term set of strategies that are not as easily correlated with the proliferation of declarations as a ‘new’ element of local government portfolios:

It does get a mention but it’s more like a cursory mention that ‘yes, we’ll have to build a more resilient system, more resilient infrastructure for the future that can withstand climate impact., blah, blah, blah’. That’s the usual rhetoric again, but how might they both interact? How might mitigation efforts and schemes interact with adaptation, for instance? Like, what are the overlaps? I don’t think we’ve considered them closely enough. (Civ1)

So I think there isn’t in terms of the coverage, the scope of those emergencies, there are gaps. I think the other thing is that some councils are just covering their own emissions and others are covering the wider area, you know, the whole council areas which is obviously much, much harder. (Civ3)

A fascinating dynamic plays out whereby realisation over the impact of climate change is used as a stimulant for taking action at the local scale, but the nature of that action is not directly linked to the direct threat climate change poses to the local borough (e.g. local impacts of climate risks) in question:

I mean they’re mainly net zero, I think the ones that I’ve read are very heavily about the zero emissions and cutting carbon. They mention climate change impact a lot, you know, sometimes in the kind of justification, the introduction, the context setting, but they don’t do an awful lot of adaptation in the stuff beyond that, in the kind of action planning or in the sort of intention sort of stuff, so we haven’t seen a lot of that. (Pol1)

Our data highlight a gap between the potential impact of climate change, which is implicit in the ‘emergency’ framing in comparison with the response, which covers mitigation rather than adaptation. Ongoing actions in one particular borough council to reduce emissions will help contribute to the global effort, and will not specifically protect that borough. Local councils could therefore gain more by enhancing their adaptation efforts that have a higher likelihood of directly benefiting them through resilience to climate risks that affect the local level. In spite of this, there is sufficient motivation to declare a climate emergency, which ultimately and primarily focuses on mitigation, over and above statutory requirements. In comparison, councils have more control over, and responsibility for, aspects of adaptation (such as emergency responses to flooding), hence the focus on mitigation as opposed to adaptation, while in the context of the global emergency is justified, requires further investigation.

### Moving forward

There was recognition from interviewees about the disconnect in language between the scale of the challenge and the nature of the response enacted (Aca1; Aca5; Pol5). This has a number of implications for how we try to correlate the declaring of an emergency with a willingness to act on climate change.

So, they call it a climate emergency but if it was actually an emergency, for instance, councils have emergency funds right? They’re not dipping into those to address it and it kind of just shows in exactly the same way the UK government has responded to Covid. It wasn’t really seen as an emergency until it was too late and then, you know, a lot of unnecessary deaths are now occurring. And this is exactly what’s happening with climate – it’s just happening over a longer period of time and it’s, you know, it’s just tragic that they don’t really get that. (Civ4)

if you don’t believe in behaviour change around this whole issue, then I think you would be very critical of any form of emergency declaration. I think without behaviour change, it’s absolutely impossible to get where we need to be. (Aca1)

In London as elsewhere (see Harvey-Scholes 2019), not all declarations are aligned with an accompanying plan or strategy, and where a plan exists, considerable variation can be identified between them:

I think in some councils they’re taken more seriously than others. So, Southwark are doing quite a good job of collaborating with their council on building a proper climate plan and they’ve done quite a reasonably good job with that and it’s kind of credible. Whereas with other ones, it’s like an aspiration or, at worst, it’s just a kind of a purely rhetorical thing of just (...) a fashionable thing to do at that big moment of climate awareness. (Civ4)

In contrast, the challenge of resourcing a post-declaration strategy looms large:

There might not be any specific money attached to it. They won’t be detailed enough scheme by scheme, policy by policy. It won’t be as detailed, but nonetheless they might do some more research and commission a few consultancies to do some research on coming up with some road maps for their net zero plans. (Civ1)

They’re in grave danger of being ignored, in lots of cases, as council resources are very stretched anyway. Most of the action plans that are on the website that have come from councils often that were declared a year or more ago are still in draft and they don’t necessarily carry with them any resources. There have been some interesting attempts to resource these better. (Pol2)

Such strategies can also appear somewhat intangible and abstract however, failing to translate into noticeable changes that benefit the local community:

I think we would want to see some quick wins being delivered, there’d be a danger of temptation to do an awful lot of analysis and baselines and modelling and all that, which some of which needs to be done but in parallel you need to actually start delivering real things that people can see. (Civ3)

So I think there’s quite a lot of scepticism about what is possible. I think there’s general recognition from the councils themselves and people more broadly, that the councils can only do so much. And there’s this huge dependency on national action. (Civ3)

Despite similarities within the declaration texts, the actions the London boroughs appear to be taking to address the climate emergency is more heterogeneous. Some boroughs appear to have not publicly taken additional climate action in the months following their declaration, while others have downplayed their responsibility for emissions, noting in their declarations that they only directly control a small proportion of emissions within the borough (e.g. Merton state 97.5% of emissions are out of their direct control: Merton, 2019). Moreover, 2030 targets set by the councils may mean different things, with some councils committing the borough to the target (e.g. Greenwich, Ealing, Hammersmith and Fulham), while others only committing to making the council carbon-neutral (e.g. Westminster). Reports and policy strategies risk falling into the same ‘rhetoric’ trap of the declarations themselves because of their failure to engage with the systemic nature of the problem:

How much of climate is embedded within that, I say, essentially the greening of existing policies, that’s one thing to look out for. (...) Many are actually in fact continuing with their existing policy without having to revise them or review them in the light of their declaration. (Civ1)

The reproduction of blueprint approaches to declarations risks undermining the need for tailored local strategies and applying these appropriately within local contexts:

my feeling personally is that there are commonalities of course between the plans, so plans should be bespoke to the council or the community. And yes, there will be massive overlaps, but that plan, in order to be effective, has to come out of the council and the communities they have to create it. (Civ5)

in the declarations, from what I’ve seen, the broad template, I think, is great. And there’s a local political commitment there. There’s sometimes the connection to local climate assemblies and the requirements to report at council meetings and stuff, which is all important. But I think the big bit that is missing is to be part of a big national voice to central government, and central government has a massive role to play to support and enable and, in some cases, require local action from local authorities. From stakeholders that are outside of local authorities but have a massive impact on emissions but they’re within the geography of the local authority, like ports, and airport, that are often privately owned, etc. (Pol6)

## Concluding remarks

By analysing the climate emergency declarations made by London borough councils, this paper has expanded our understanding of what such emergency declarations by local governments actually *are* and how they came about at the time that they did and, most importantly, provided solid conceptual grounding for the asking the question on many people lips: what comes next? Having framed climate change’s status as a local governance issue using the concept of ‘slow emergency’, we identified four journeys to declaration (made actively from above, passively from above, actively from below, and passively from across) each demonstrating the roles played by forces both within and beyond the control of local governments.

We subsequently identified three interwoven purposes seen to be played by these declarations, each offering their own unique insight into the role of CEDs moving forward (statements of intent, acting as a political gesture and stimulating local action). These also, however, offer important insight into the multifaceted resources required to deliver on the declarations and the entwined and heterogeneous web of motives from across London borough councils. In summary, these findings demonstrate that the emergency declarations themselves serve as a form of intervention by local governments and can be considered as a means of taking action on climate change in and of themselves as much as seeing them as designed to encourage action in the future.

It is perhaps, therefore, what is missing from these declarations that is most illuminating for the question of what comes next. Both in terms of where declarations are missing entirely (in the form of boroughs that have actively resisted the pressure to declare) and in terms of what is missing from within the declarations of those who *have.* And while we believe that both of these areas will offer productive avenues for future research engagement, we want here to reflect on what these gaps might tell us about some of the practical challenges facing local government moving forward. Challenges which now exist not only in the shadow of widespread climate emergency declarations (now at national and international scales as well as local) but also of the COVID-19 pandemic and the emergency regimes of governance that *it* has ushered in.

Local authorities are rapidly being ushered into an era of needing to plan as much *with* climate change (by virtue of its increasing presence on the governance agenda) as needing to plan *for* it (Dujardin, 2020). As new roles, responsibilities and opportunities arise to drive forward action on climate change at the local level questions about the effectiveness of emergency declarations as tools for governance and their ongoing impact therefore need to be expanded beyond plans and strategies to also include their impact on local democracy (Asayama et al. 2019) and on under-resourced, overburdened local authorities (Porter et al. 2015). Rather than closing down freedoms and democracy as associated with more ‘traditional’ emergency governance regimes (so viscerally demonstrated by national government responses to COVID-19), perhaps a state of emergency declared at the local level can lead to a reinvigoration and re-politicisation of climate change action from the ‘bottom-up’. Indeed, in dealing with the challenges posed by the recent pandemic, local government has had to adapt and evolve its role to support communities isolated by the restrictive measures enacted by powerful national governments.

Thinking forward to the next stage and the prospective implementation of climate action plans emerging from the declarations, then, it is vital that we recognise the disconnect between where the sense of urgency is being communicated *from* and where the capacities lie to respond to this urgency. In some areas across the UK (e.g. cities of Oxford and Leeds), declarations have become vehicles for climate-related initiatives accompanied by encouraging budgets. However, the stated intent for ambitious climate action is thwarted in some councils by the lack of resource, skill and capacity to deliver and here runs the risk of the declarations becoming symbolic acts rather than platforms upon which further action is delivered. We therefore need a real phase-change in how expectations placed on the local scale to take action are serviced with resources (both financial and knowledge-based). Without this, the emergency declarations risk oversimplifying what is currently possible amidst a complex and ‘messy’ landscape of local climate governance (Castan Broto, 2020).

Finally, and perhaps most importantly, in their intent, the emergency declarations by local governments remain heavily bound up in a discourse about the pursuit of a reduction in CO_2_ emissions. The need to reduce emissions (mitigation) is arguably where the notion of an emergency and the urgency of action are currently finding most salience in the UK. As we have demonstrated in this paper, however, it is perhaps with adaptation that a tangible set of feedback benefits can be identified, and local councils can play a more prominent role in owning the benefits of climate action locally. Mitigation and adaptation offer us a broader rubric through which to interpret the emergency declarations of local authorities in two very different ways: firstly, as a call for collective action in national and international power circles and as an outward facing voice for local communities and, secondly, as a call to action *within* their jurisdictions and an inward facing starting gun for the pursuit of more climate aware, sustainable, resilient places for communities increasingly threatened by climate change.

## References

[CR1] Agamben G (2008) State of exception (translated by Kevin Attell). Chicago: University of Chicago press

[CR2] Allen M (2019) Why protestors should be wary of ‘12 years to climate breakdown’ rhetoric. The Conversation. Available online at: https://theconversation.com/why-protesters-should-be-wary-of-12-years-to-climate-breakdown-rhetoric-115489

[CR3] Allin S (2019) Barnet councillors fail to back climate goal. 31 July 2019. Available online https://www.times-series.co.uk/news/17807145.barnet-councillors-fail-back-climate-goal/

[CR4] Anderson B, Grove K, Rickards L, Kearnes M (2019). Slow emergencies: temporality and the racialized biopolitics of emergency governance. Prog Hum Geogr.

[CR5] Angelo H, Wachsmuth D (2020). Why does everyone think cities can save the planet?. Urban Stud.

[CR6] Asayama S, Bellamy R, Geden O, Pearce W, Hulme M (2019). Why setting a climate deadline is dangerous. Nat Clim Chang.

[CR7] Bandt A (2009). Had we but world enough and time (reconsidering ‘emergency’). Aust Fem Law J.

[CR8] Bull T (2019) Bromley sets out target of being carbon neutral by 2029. Bromley Borough News, July 2019. Available online http://www.bromleyboroughnews.co.uk/article.cfm?id=131302&headline=Bromley+sets+outs+target+of+being+carbon+neutral+by+2029&sectionIs=News&searchyear=2019

[CR9] Burford R, King J (2020) Barking and Dagenham council declares a climate emergency. Barking and Dagenham Post, 5 February 2020, Available online https://www.barkinganddagenhampost.co.uk/news/environment/barking-and-dagenham-climate-emergency-1-6499178

[CR10] Calhoun C (2010) “The idea of emergency: humanitarian action and global (dis)order”, in Fassin D, Pandolfi M., Contemporary states of Emergency

[CR11] Castan Broto V (2020). Climate change politics and the urban contexts of messy governmentalities. Territ Politics Gov.

[CR12] Climate Alliance (2019) Climate emergency resolution template. Available online https://www.climatealliance.org/fileadmin/Inhalte/2_Municipalities/Climate_Emergency/2019-05_Climate_EmergencyTemplate.pdf

[CR13] Creasy A, Lane M, Owen A et al (2021) Representing place; how urgency trumps justice in experimental climate governance for cities. Politics and Governance 9(2):64–75. 10.17645/pag.v9i2.3794

[CR14] Cretney R, Nissen S (2019) Climate politics ten years from Copenhagen: activism, emergencies, and possibilities. Women talking politics. New Zealand Political Studies Association. https://nzpsa.co.nz/resources/Documents/WTP/Women%20Talking%20Politics%202019.pdf#page=15

[CR15] Davidson K, Briggs J, Nolan E, Bush J, Hakansson I, Moloney S (2020) The making of a climate emergency response: examining the attributes of climate emergency plans. Urban Clim 33(100666)

[CR16] Di Gregorio M, Fatorelli L, Paavola J, Locatelli B, Pramova E, Nurrochmat DR, May PH, Brockhaus M, Sari IM, Kusumadewi SD (2019). Multi-level governance and power in climate change policy networks. Glob Environ Chang.

[CR17] Dujardin S (2020). Planning with climate change? A poststructuralist approach to climate change adaptation. Ann Am Assoc Geogr.

[CR18] Extinction Rebellion (2018a) The Extinction Rebellion climate factsheet for Rebels - Extinction Rebellion. Extinction Rebellion. https://rebellion.earth/2018/11/17/the-climate-factsheet-for-rebels/

[CR19] Extinction Rebellion (2018b) Extinction Rebellion letter to BBC: ‘tell the truth on climate emergency’ - protests this Friday - Extinction Rebellion. Extinction Rebellion. https://rebellion.earth/2018/12/17/extinction-rebellion-letter-to-bbc-tell-the-truth-on-climate-emergency-protests-this-friday/

[CR20] Green World (2020) Climate emergency declarations held back by central government. Available online at https://greenworld.org.uk/article/climate-emergency-declarations-held-back-central-government

[CR21] Hackney Council (2019) Hackney council pledges to reach net zero emissions by 2040. 28 June 2019. Available online https://news.hackney.gov.uk/hackney-council-pledges-to-reach-net-zero-emissions-by-2040/

[CR22] Harvey-Scholes, C. (2019). Climate emergency declarations accelerating decarbonisation? What 249 UK examples can tell us. IGov and University of Exeter. Available online at: https://projects.exeter.ac.uk/igov/new-thinking-climate-emergency-declarations-accelerating-decarbonisation/

[CR23] Hulme M (2019) Is it too late (to stop dangerous climate change)? An editorial. Wires Climate Change 11(1). 10.1002/wcc.619

[CR24] Humphreys S (2006). Legalizing lawlessness: on Giorgio Agamben’s state of exception. Eur J Int Law.

[CR25] IPCC (2019a) Special report: Global warming of 1.5°C. Retrieved from: https://www.ipcc.ch/sr15/

[CR26] IPCC (2019b) History and general information. IPCC. Retrieved from: https://www.ipcc.ch/2019/

[CR27] Jordan A, Huitema D, van Asselt H, Forster J (eds) (2018). Governing climate change: polycentrically setting the scene. Governing Climate Change. Cambridge University Press 10.1017/9781108284646

[CR28] Keohane RO, Victor DG (2011) The regime complex for climate change. Perspectives on Politics 9(1):7–23. 10.1017/S1537592710004068

[CR29] Lindsay B (2010) Climate of exception: what might a ‘climate emergency’ mean in law? Fed Law Rev 38(2):255–281. 10.22145/flr.38.2.4

[CR30] Nixon R (2011) Introduction. In slow violence and the environmentalism of the poor (pp. 1–44). Harvard University Press; JSTOR. https://www.jstor.org/stable/j.ctt2jbsgw

[CR31] Oxford English Dictionary (2020) Climate emergency. Retrieved from: https://www.oxfordlearnersdictionaries.com/definition/english/climate-emergency

[CR32] Porter JJ, Demeritt D, Dessai S (2015). The right stuff? Informing adaptation to climate change in British local government. Glob Environ Chang.

[CR33] Ripple W, Wolf C, Newsome T, Barnard P, Moomaw W (2020). World scientists’ warning of a climate emergency. BioScience.

[CR34] Roach A (2019) No climate emergency for Havering but councillors will review environmental protection policies. Romford Recorder, 12 July 2019. Available online https://www.romfordrecorder.co.uk/news/environment/havering-votes-against-declaring-climate-emergency-1-6155423

[CR35] Rode P (2019) Climate emergency and cities: an urban-led mobilisation? LSE Cities Discussion Papers. Retrieved from: http://www.lse.ac.uk/cities/Assets/Documents/Climate-Emergency-and-Cities-An-urban-led-mobilisation.pdf

[CR36] Schlosberg D, Collins L, Niemayer S (2017). Adaptation policy and community discourse: risk, vulnerability, and just transformation. Environ Politics.

[CR37] Sotto D, Philippi A, Yigitcanlar T, Kamruzzaman M (2019). Aligning urban policy with climate action in the Global South: are Brazilian cities considering climate emergency in local planning practice?. Energies.

[CR38] Spratt D, Sutton P (2008) Climate 'code red'. Fitzroy: friends of the Earth

[CR39] Sutton P (2017) Local first implementation. Why a strong climate declaration is needed – at the local government level – and what it can do. RSTi. Available http://www.green-innovations.asn.au/RSTI/Local=first-implementation_local-govt.pdf

[CR40] UNEP (2019) The UN environment programme at the climate action summit. UNEP. Retrieved from: https://www.unenvironment.org/unga/our-position/unep-climate-action-summit

[CR41] UNEP (2020a) Facts about the climate emergency. UNEP. Retrieved from: https://www.unenvironment.org/explore-topics/climate-change/facts-about-climate-emergency

[CR42] UNEP (2020b) Perfect storm: when climate change stokes wildfires, marine heatwaves and biodiversity loss. UNEP. Retrieved from: https://www.unenvironment.org/news-and-stories/story/perfect-storm-when-climate-change-stokes-wildfires-marine-heatwaves-and

[CR43] UNEP (2020c) The UN Environment Programme and the climate emergency. UNEP - UN Environment Programme. https://www.unenvironment.org/unga/our-position/unep-and-climate-emergency

[CR44] Van der Heijden, J. (2018). City and subnational governance. In A. Jordan, D. Huitema, H. Van Asselt, & J. Forster (Eds.), Governing Climate Change: Polycentricity in Action? (pp81-96). Cambridge: Cambridge University Press. 10.1017/9781108284646.006

[CR45] Van der Heijden J (2019). Studying urban climate governance: Where to begin, what to look for, and how to make a meaningful contribution to scholarship and practice. Earth System Governance.

[CR46] Zhou N (2019) Oxford Dictionaries declares “climate emergency” the word of 2019. The Guardian. Retrieved from: https://www.theguardian.com/environment/2019/nov/21/oxford-dictionaries-declares-climate-emergency-the-word-of-2019

